# Supplementary material for: Temporal loudness weights: Primacy effects, loudness dominance and their interaction
Source: PLoS One. 2021 Dec 23;16(12):e0261001. doi: 10.1371/journal.pone.0261001 (PMC8699981; doi:10.1371/journal.pone.0261001)
Supplement: Attachment — Submitted filename: Response_Fischenich_et_al_Loudness_Reviewer1_2.pdf [file pone.0261001.s001.pdf]

PONE-D-21-12847

Temporal loudness weights: Primacy effects, loudness dominance and their interaction

Reviewer #1:

The paper describes three experiments on loudness perception in groups of 8-10 listeners. The experiments were similarly designed. The listeners had to judge on the loudness of a set of stimuli (wbn) where the level of one of the stimuli (first, third or seventh) was lowered or increased. Gap duration between the single stimuli and duration of the stimuli varied in the experiments. It was found that attenuation has less influence on loudness than amplification. The primacy effect seems to be the dominant effect.

In summary, the paper is mostly correct. The presented experiments are appropriate to testing for the hypotheses. However, the experiments should be better justified in the introduction. In the current version it looks like a potpourri without a strong connection. Conclusions are mostly justified by the results. However, writing style is not appropriate and the readability is very difficult and somewhat tedious. I would like to encourage the authors to rewrite the manuscript and to provide a better guidance for the reader. For example the hypotheses should be presented clearer.

Thank you for this comment. Each experiment was already motivated in the introduction, but we agree that some more details may be helpful. The introduction was rewritten accordingly. To provide a better guidance for the reader we added some additional passages and at the end of each experiment, to present the motivation of the experiments and the research question that is evaluated within each of the experiments. We also rephrased those parts of the manuscript, where the sentences were extremely long which may have hampered the reading of the manuscript.. Furthermore, because of the responses from Reviewer #2, we now stated more explicitly whether we refer to temporal weights or weight factors when we report and discuss effects and their size. We hope that the above reported changes led to a better guidance for the reader and improved the readability of the whole manuscript.

E.g. the figures illustrating the stimulus settings could be increased: Why do you show example levels? It would be more intuitive to provide mean values (lines and standard deviations) ‟ The arbitrary bars can be omitted.

The figure below shows a figure with means and SDs, as suggested. In our view, this does not easily communicate the fact that the segment levels varied within a trial. The SDs were identical for each segment, i.e., this is also not an information that needs to be provided in the figure. We would thus prefer to keep the original version of the trial figures, but if you feel that the alternative version is better, we will use the latter version.

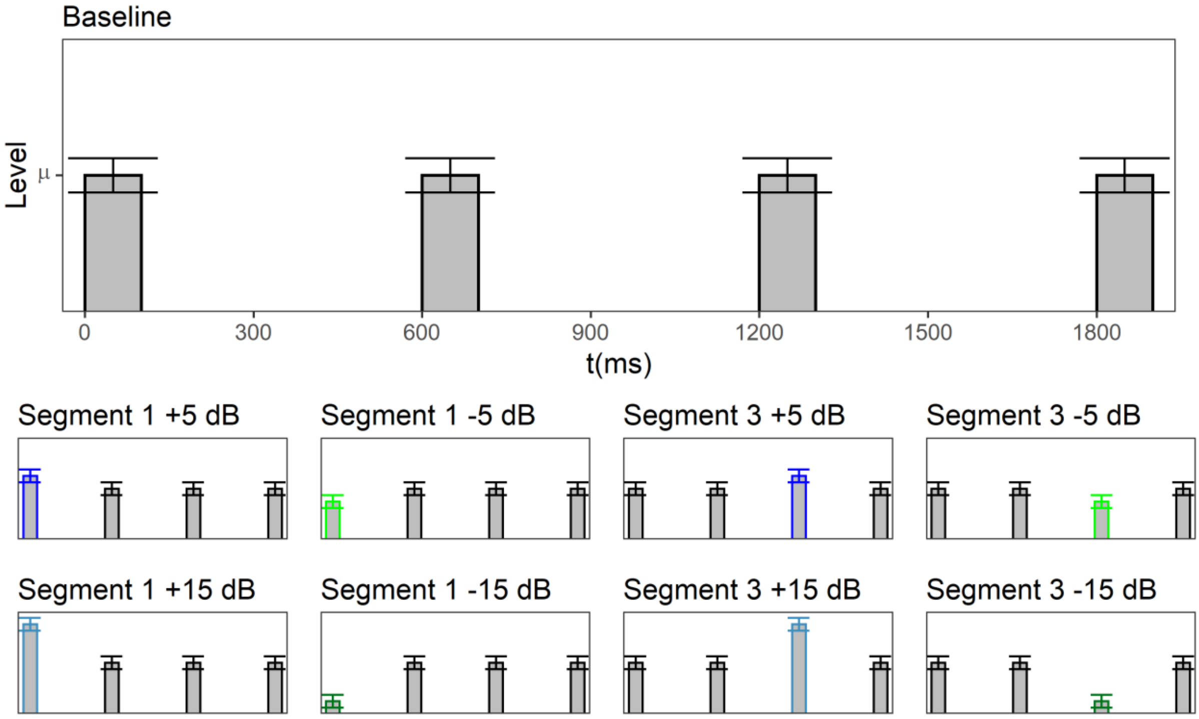

It is hard to understand help
Please justify the use of different mean levels. What is the meaning of the chosen values (56.125 dB SPL)?

This is approach is not uncommon in this type of experiments (e.g. Pedersen B, Ellermeier W. Temporal weights in the level discrimination of time-varying sounds. J Acoust Soc Am. 2008;123(2):963-72; Ponsot E, Susini P, Saint Pierre G, Meunier S. Temporal loudness weights for sounds with increasing and decreasing intensity profiles. J Acoust Soc Am. 2013;134(4):EL321-EL6.). The motivation is twofold: a) they make the task objective (because we can then classify a response as correct when, e.g., the segment levels were drawn from the distribution with the higher mean and the listener responded that this was a "louder" stimulus) and b) presenting two different mean levels allowed us to adjust the difficulty of the task. By increasing or decreasing the level difference between the two distributions' means, the task gets easier or more difficult. For our experiment, we selected a level difference at which the listeners were able to respond with roughly 70% correct. The values you refer to are simply the means of the two level distributions.

I am not familiar with the concept of intensity resolution. However, I would expect another measurement to quantify intensity resolution.

What we argue is that based on previous research, non-simultaneous masking effects on the intensity resolution is likely to affect the temporal weights in some of the conditions / experiments. It would indeed be an interesting approach for future experiments to explicitly measure the intensity resolution for "isolated" segments in suitable control conditions and to compare the measured intensity resolution for the different segments to the pattern of temporal weights, to evaluate to which extent the temporal weights are correlated with the intensity resolution. In our view, this is beyond the scope pf the present study but would certainly be an interesting research question of a new project.

To clarify this point, we now added the following passage to the discussion section of the manuscript:

Furthermore, one has to note that we did not measure the intensity resolution for specific segments in the present study, but based our reasoning on previous research concerning non-simultaneous masking effects on the intensity resolution. It would be an interesting approach for future experiments to explicitly measure the intensity resolution for "isolated" segments in suitable control conditions and to compare the measured intensity resolution for the different segments to the pattern of temporal weights, to evaluate to which extent the temporal weights are correlated with the intensity resolution.

What was the meaning of different definitions for weights of attenuation and amplification (Eq.2 and 3) Is this the caus for postive weights for attenuation?

The temporal weights were defined in the same way for a given sound segment that was attenuated or amplified relative to the remaining segments. We think you refer to the *weight factors*. The motivation for defining them differently for amplified and attenuated segments was to be able to compare the absolute value of the change in weight caused by attenuation (which results in a reduction in the weight assigned to the segment) and amplification (which results in an increase of the weight assigned to the segment) on the same scale. We stated in the original manuscript within the results section of Experiment 1, when the analyses based on the weight factors were first introduced:

Note that these weight factors were defined so that both an increase in weight due to an amplification and a decrease in weight due to an attenuation of a given segment corresponded to a weight factor larger than 1.0 (see Eqs. 1 and 2 above).

In addition, we added the following text to further clarify this point:

In order to facilitate the comparison of the effects of attenuation and amplification on the weight assigned to the segment changed in level, we defined the weight factor for the case of attenuated segments so that a reduction in weight corresponded to a weight factor larger than 1.0. […] Thus, if for instance an amplification by 10 dB caused an increase in the weight assigned to the amplified segment by a factor of 3 relative to the segment weight in the baseline condition, and an attenuation by 10 dB caused a decrease in weight again by a factor of 3, then with the above definitions of the weight factors, the weight factor would be 3.0 for amplification as well as for attenuation. Thus, using this definition, the effect of amplification or attenuation on the weights is in this case the same for the same dB change (weight change by a factor of 3), although of course in opposite direction (increase when amplified, decrease when attenuated).

We now provide this explanation for this definition directly after the definition of the weight factor for amplified segments in the section “Data Analysis”.

Why do you explain ROC analysis? I am not sure whether this is rather necessary. D-prime and ROC-areas are very similar in their meaning. Maybe a scetch of the data analysis and a more extensive data representation (if necessary nin the appendix) would help.

Thank you for raising this point. As you pointed out correctly, the two statistics are indeed very similar when applied to the *same aspect* of the data. However, as we explain in the paper, the AUC values we report measure the predictive accuracy of the logistic regression models and can thus be viewed as a goodness-of-fit measure (cf. Hosmer, D. W., and Lemeshow, S., 2000, Applied Logistic Regression, Wiley, New York; and for a detailed explanation see Dittrich & Oberfeld, 2008, *A comparison of the temporal weighting of annoyance and loudness*, J. ACOUST. SOC. AM., p. 3171). The *d´* values, on the other hand, measure the performance of the listeners in the perceptual tasks. Thus, the reported AUC values and the reported *d´* values address two different aspects of the data. The decision model used for the data analyses is now described explicitly in the paper.

I recommend a better description of the results and an extensive explanation of the derivation of the weights.

We now explicitly describe the decision model used for the analyses in the section “Data Analysis” within the paper.

The writing style needs enhancement. For example, it is confusing when you write about Exp 2 (l303) and start with "Experiment 1 …."

Thank you for this comment, we changed the introduction to Experiment 2. Also, as stated above and below, we changed some of the long sentences, referred to the statistics more explicitly and rewrote

some of the passages that motivate the experiments and discuss the results.

Monster sentences like “For a level fluctuating sound, the probability that a given temporal segment …” should be avoided. It is quite hard to understand this .

We agree. We carefully checked the manuscript and rewrote overly complicated sentences.

Reviewer #2

The study by Fischenich and co-workers investigates loudness judgement of human listeners, in particular the primacy effect and its possible interaction with loudness dominance of temporal components within a sound. The authors report no such interaction for the attenuation of temporal sound components, whereas for amplification an interaction seems likely.
The study is well conducted, methods and statistics are sound, although some parts of the text are confusing (cf. below).

Specific comments

Major:
How the size of the effects of attenuation vs. amplification is described in the text is often confusing. For example:
Line 299: “the effect of attenuation was considerably larger than the effect of amplification” does not seem to reflect the figure, but it does fit to the table.
Another example: line 581f (“the effect of amplification was larger than the effect of attenuation”) seems to contradict line 701f (“…stronger effect of attenuation compared to amplification observed in the experiments”). To avoid such confusion, whenever such comparisons of effects of attenuation and amplification are made throughout the text, please describe exactly what effect is referred to.

Thank you for pointing this out, we now always make it explicit when we refer to the weights factors rather than to the normalized weights.

Minor:

In the Data Analysis section the decision model is described (line 178ff) and the interpretation of the whole study is based on that model, but this is not discussed in the Discussion section. What impact would alternative models for loudness judgement, e.g. a rating based on the loudest segment only, have on the interpretation of the data? This should at least shortly be discussed.

Thank you for raising this point.

Alternative models based on the maximum or median or 90th percentile of the loudness calculated with the time varying partial loudness model by Glasberg and Moore (2005) were discussed in an earlier study (Fischenich A, Hots J, Verhey J, Oberfeld D. Temporal weights in loudness: Investigation of the effects of background noise and sound level. PloS one. 2019;14(11):e0223075.). The results showed that models based on the weighted segment levels provided the best prediction of the data compared to those other models. Furthermore, the models did not predict a primacy effect.

Following your suggestion, we fitted logistic regression models to the data of Experiment 1 that contained only the maximum segment level within each trial as a predictor (i.e., the level of the loudest segment, as you suggested). We did this again for each participant within each condition separately. The average predictive power in terms of the AUC of those models was lower than that of the “segment-model” with a mean AUC of 0.72 (*SD* = 0.07, range 0.57 - 0.87).

Equally important, please note that for the present study, a model based on only the loudest segment would result in an exclusive weight on the *amplified segments* for the conditions in which the segments received a 15 dB amplification, because the sound level exceeded the sound level of all other segments on virtually all of the trials. However, as can be seen in Figure 4, most of the weights on the unamplified segments still differed from zero. For an attenuation of 15 dB, the "maximum" model would predict zero weights, which is indeed in line with our results. However, the "maximum" model would not predict the primacy effect on the unattenuated segments, as it was observed in Experiment 2, and also not a primacy effect in the baseline condition (as observed for the contiguous sounds).

We added a short passage to the discussion that addresses this point. It reads:

The results discussed so far were based on the decision model described within the section "Data Analysis". We also investigated a different decision model that was solely based on the sound level of the loudest segment within each trial. For the data of Experiment 1, the average predictive power of such a model in terms of the AUC was 0.72 (SD = 0.07, range 0.57 - 0.87) and thus significantly lower than for the model based on a weighted average of the segment levels. Furthermore, a model based on only the loudest segment would result in an exclusive weight on the amplified segments for the conditions in which the segments received a 15 dB amplification, because the sound level exceeded the sound level of all other segments on virtually all of the trials. However, as shown in Figure 4, most of the weights on the unamplified segments still differed from zero. For an attenuation of 15 dB, the "maximum" model would predict zero weights, which is indeed in line with our results. However, the "maximum" model would not predict the primacy effect on the unattenuated segments, as it was observed in Experiment 2, and also not a primacy effect in the baseline condition (as observed for the contiguous sounds).

Line 375: Remove extra full stop.
Changed as suggested

Line 396f: … with amplifying the beginning of the sound resulting in smaller weight factors compared to attenuation of the middle position.

Changed as suggested

Line 414f: With respect to Fig. 4, this seems to be true for the third segment, but not for the 7th.

For the weight change it is true for both segments, but we agree with your point raised earlier (in the major points) that this was not always clear. We therefore now explicitly mention that changes in the weight factors are described.
